# Serum amyloid A predisposes inflammatory tumor microenvironment in triple negative breast cancer

**DOI:** 10.18632/oncotarget.26566

**Published:** 2019-01-11

**Authors:** Rosa Mistica C. Ignacio, Carla R. Gibbs, Soohyun Kim, Eun-Sook Lee, Samuel E. Adunyah, Deok-Soo Son

**Affiliations:** ^1^ Department of Biochemistry, Cancer Biology, Neuroscience and Pharmacology, Meharry Medical College, Nashville, TN, USA; ^2^ Department of Veterinary Sciences, College of Veterinary Medicine, Kon-Kuk University, Seoul, Republic of Korea; ^3^ Department of Pharmaceutical Sciences, College of Pharmacy, Florida A&M University, Tallahassee, FL, USA

**Keywords:** serum amyloid A, proinflammatory, tumor microenvironment, triple negative breast cancer, interleukin-1β

## Abstract

Acute-phase proteins (APPs) are associated with a variety of disorders such as infection, inflammatory diseases, and cancers. The signature profile of APPs in breast cancer (BC) is poorly understood. Here, we identified serum amyloid A (SAA) for proinflammatory predisposition in BC through the signature profiles of APPs, interleukin (IL) and tumor necrosis factor (TNF) superfamily using publicly available datasets of tumor samples and cell lines. Triple-negative breast cancer (TNBC) subtype highly expressed *SAA1/2* compared to HER2, luminal A (LA) and luminal B (LB) subtypes. *IL1A*, *IL1B*, *IL8*/*CXCL8*, *IL32* and *IL27RA* in IL superfamily and *CD70*, *TNFSF9* and *TNFRSF21* in TNF superfamily were highly expressed in TNBC compared to other subtypes. SAA is restrictedly regulated by nuclear factor (NF)-κB and IL-1β, an NF-κB activator highly expressed in TNBC, increased the promoter activity of SAA1 in human TNBC MDA-MB231 cells. Interestingly, two κB-sites contained in SAA1 promoter were involved, and the proximal region (−96/−87) was more critical than the distal site (−288/−279) in regulating IL-1β-induced SAA1. Among the SAA receptors, *TLR1* and *TLR2* were highly expressed in TNBC. Cu-CPT22, TLR1/2 antagonist, abrogated IL-1β-induced SAA1 promoter activity. In addition, SAA1 induced IL8/CXCL8 promoter activity, which was partially reduced by Cu-CPT22. Notably, *SAA1/2*, *TLR2* and *IL8/CXCL8* were associated with a poor overall survival in mesenchymal-like TNBC. Taken together, IL-1-induced SAA via NF-κB-mediated signaling could potentiate an inflammatory burden, leading to cancer progression and high mortality in TNBC patients.

## INTRODUCTION

Serum am4yloid A (SAA), an acute phase protein (APP), is mainly produced in the liver, while its extrahepatic synthesis has been reported in skin [1], in atherosclerotic lesions [2], in synovial tissues [3], in adipocytes and smooth muscle cells [4, 5]. The human SAA acute phase protein family contains four different isoforms namely, SAA1, SAA2, SAA3 and SAA4. Both SAA1 and SAA2 show high degree of homology in the mRNA and the protein sequences which are hard to be identified [6]. SAA3 is a pseudogene categorized as a non-coding RNA [7], while SAA4 is constitutively expressed as a non-inducible protein [8, 9]. On the other hand, the synthesis of SAA1 and SAA2 is inducible under inflammatory conditions [9], such as inflammation, trauma and infection, increasing to several hundredfold [10, 11].

SAA is a family of apolipoproteins associated with high-density lipoprotein, playing a role in AA-type amyloidosis and cholesterol metabolism and transport [12, 13]. Emerging studies of SAA have been implicated in promoting cellular migration [14, 15], augmenting cytokines and chemokines expression [16–18], stimulating angiogenesis [19, 20], inducing the transcription factor nuclear factor (NF)-κB, and activating signaling pathways, such as ERK1/2, p38 and JNK [17, 20–23]. Cumulative studies have shown that SAA is upregulated in a wide range of malignancies, such as lung [24–26], ovarian [23, 27], pancreatic [28, 29], prostate [30], uterine serous papillary [31] and renal cell [32] carcinomas. We have reported that SAA is preferentially localized to ovarian epithelial cells and the thecal-interstitial layers compared to granulosal cell layers in a mouse ovary [33], and ovarian carcinoma show tumor necrosis factor α (TNF)-induced SAA production [23]. However, expression of SAA in breast cancer (BC) is poorly understood.

BC is the most frequently diagnosed cancer and the second most common cause of cancer deaths in women in the US [34]. One probable reason for this is that we lack a complete picture of the biologic heterogeneity of this disease. BC is divided into 4 intrinsic subtypes with a molecular basis as follows: luminal A (LA), luminal B (LB), human epidermal growth factor receptor 2 (HER2)-enriched, and basal-like (BL) BC [35–38]. Triple negative breast cancer (TNBC), lacking estrogen receptor (ER), progesterone receptor (PR) and HER2, is known to be the most heterogeneous and comprises largely of the basal-like subtype [39]. LA-BC is characterized by ER or PR positive but HER2 negative, while LB-BC presents with ER or PR and HER2 positive [40]. The HER2-enriched subtype is characterized by HER2 positive, both ER and PR negative, and high expression of proliferation-related genes [40]. The BL subtype includes plenty of TNBC and high expression of EGFR and proliferation-related genes [36, 41]. Furthermore, TNBC, the most heterogeneous BC, were defined into several subtypes namely, basal-like 1 and 2 (BL1, BL2), mesenchymal-like (ML) and luminal androgen (LAR) [42]. BL-TNBC subtype is characterized by highly activated cell cycle and DNA [43]. ML-TNBC is associated with poor prognosis due to enhanced epithelial-to-mesenchymal transition (EMT) [43] and elevated expression of genes involved in growth factor pathways [42, 44]. LAR-TNBC expresses differentially the estrogen/androgen metabolism pathways and is driven by androgen signaling [43, 45]. BL-BC representing TNBC is of particular interest because of aggressiveness, early pattern of metastasis, greater size of tumor, and lack of well-defined therapeutic target sites due to ER-/PR-/HER2-negative status [39, 46]. Most patients with TNBC have experienced higher rate of distant recurrence compared to patients with other BC subtypes, requiring identification of molecular drivers for TNBC.

To date, the signature profile of APPs in BC is not well defined. Here, we analyzed the signature profiles of APPs, interleukin (IL) family and TNF superfamily using publicly available datasets of breast tumor samples and cell lines. Based on the results analyzed, we found SAA as a main factor for proinflammatory predisposition in TNBC and proposed IL-1-induced SAA via NF-κB-mediated signaling as a molecular driver for the aggressiveness of TNBC.

## RESULTS

### *SAA1* and *SAA2* APPs are predominantly expressed in human BL-BC subtype and TNBC cells

**Figure 1:**
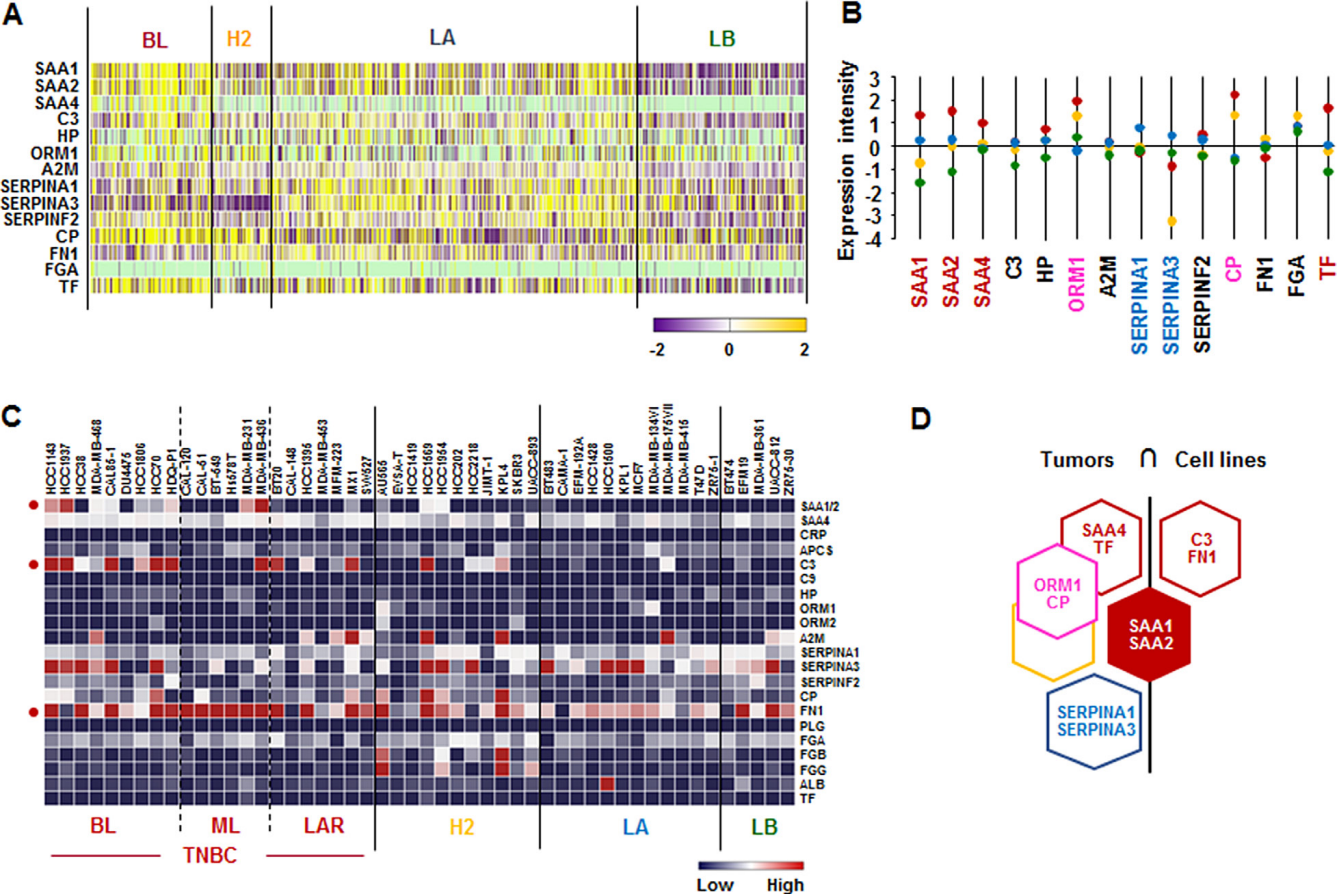
Acute-phase protein signatures in BC tissues and cell lines (**A**) Heatmap for APP expression profiles in human BC tissues from TCGA-based dataset using Gitools 2.3.1. (**B**) Statistical analysis of APP expression intensity in human BC tissues. (**C**) Heatmap for RNA expression levels of APPs based on analysis of GEO dataset (Accession: GSE12777) with 51 human BC cell lines using Gitools 2.3.1. (**D**) Intersection of APP signature between human BC tissues and cell lines. Red, yellow, blue and green dots specify high expression levels in BL-, HER2 (H2)-, LA- and LB-BC subtypes, respectively. Pink letters specify high expression levels in both BL- and HER2-BC subtypes. ML; mesenchymal-like, LAR; luminal androgen receptor and TNBC; triple-negative breast cancer.

We investigated the APP signature in human breast tumor tissues and cell lines. We used The Human Cancer Genome Atlas (TCGA)-based dataset for human BC tissues to define the APP signature in breast tumor heterogeneity. Analysis of TCGA-based dataset by Gitools 2.3.1 revealed the following dominant APP signature: BL-BC subtype representing TNBC highly expressed *SAA1*, *SAA2*, *SAA4* and *TF*. Both BL and HER2 subtypes highly expressed *ORM1* and *CP* (Figure 1A and 1B), while LA subtype highly expressed *SERPINA1* and *SERPINA2* (Figure 1A and 1B). In addition, the analysis of the National Center for Biotechnology Information (NCBI) Gene Expression Omnibus (GEO) dataset on 51 human BC cell lines revealed the following signature of APP: *SAA1/2* was highly expressed in both BL- and ML-TNBC; *C3* and *FN1* were predominantly expressed in BL-and ML-TNBC cells, respectively (Figure 1C and Supplementary Figure 1). Based on the intersection of APP signature between human BC tissues and cell lines (Figure 1D) to exclude the tumor heterogeneity, *SAA1* and *SAA2* were highly expressed both TNBC tissues and cell lines (Figure 1D).

### *IL1A, IL1B, IL8/CXCL8* and *IL32* are highly expressed in human BL-BC subtype and TNBC cells

**Figure 2:**
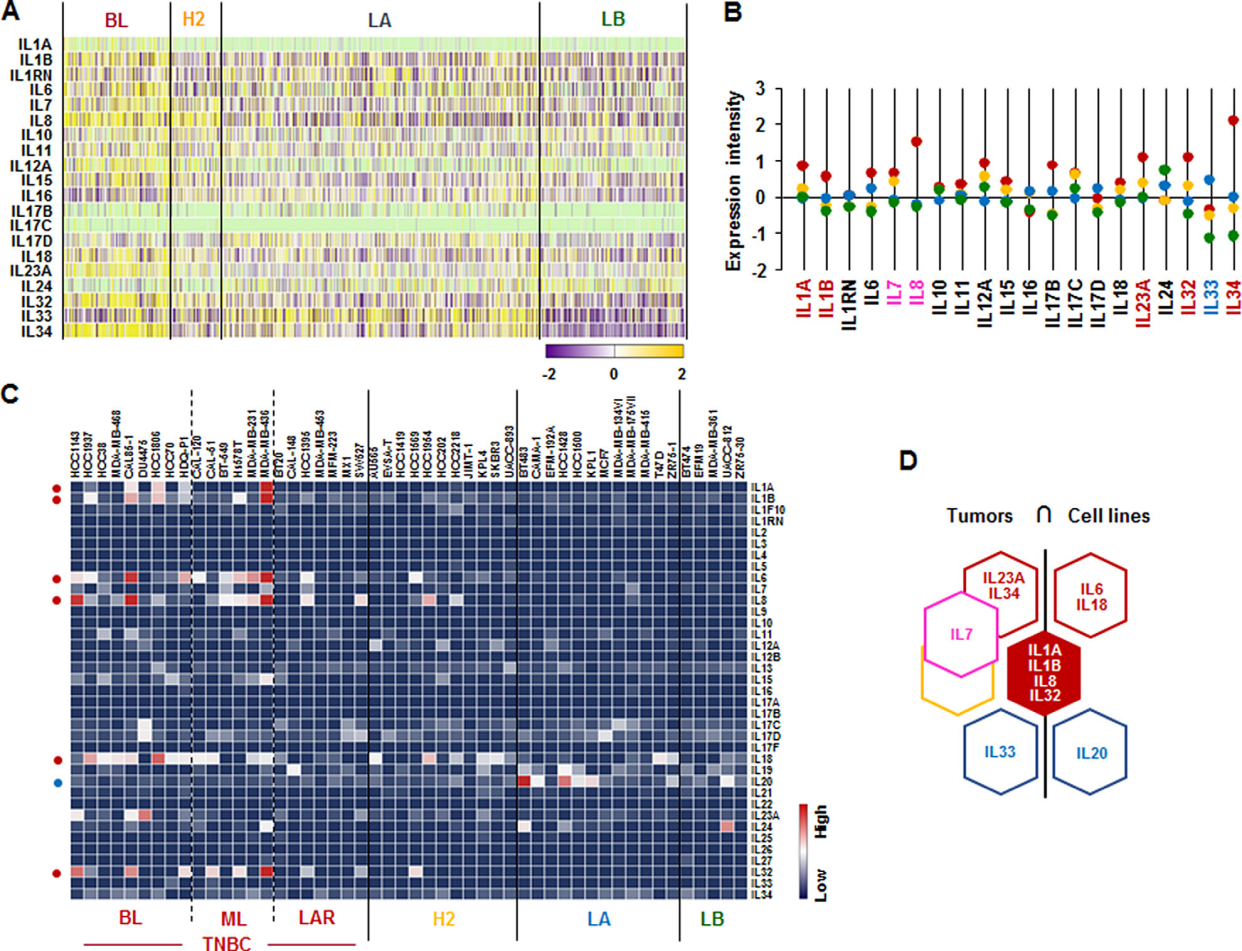
Interleukin superfamily signatures in BC tissues and cell lines (**A**) Heatmap for IL superfamily expression profiles in human BC tissues from TCGA-based dataset using Gitools 2.3.1. (**B**) Statistical analysis of IL superfamily expression intensity in human BC tissues. (**C**) Heatmap for RNA expression levels of IL superfamily based on analysis of GEO dataset (Accession: GSE12777) with 51 human BC cell lines using Gitools 2.3.1. (**D**) Intersection of IL superfamily signature between human BC tissues and cell lines. Red, yellow, blue and green dots specify high expression levels in BL-, HER2 (H2)-, LA- and LB-BC subtypes, respectively. Pink letters specify high expression levels in both BL- and HER2-BC subtypes. ML; mesenchymal-like, LAR; luminal androgen receptor and TNBC; triple-negative breast cancer.

We analyzed the IL superfamily signature in human breast tumor tissues and cell lines. We also used TCGA-based dataset for human BC tissues and NCBI-GEO dataset for 51 human BC cell lines. Particularly, BL-BC subtype representing TNBC highly expressed *IL1A*, *IL1B*, *IL23A*, *IL32* and *IL34* (Figure 2A and 2B). Both BL- and HER2-BC subtypes predominantly expressed *IL7* and *IL8*, while LA-BC subtype highly expressed *IL33* (Figure 2A and 2B). In addition, both BL- and ML-TNBC human cell lines highly expressed *IL1A*, *IL1B*, *IL6*, *IL8/CXCL8* and *IL32*, BL-TNBC cell lines highly expressed IL18, and LA cell lines dominantly expressed *IL20* (Figure 2C and Supplementary Figure 2). Based on the intersection of interleukin superfamily signature between human BC tissues and cell lines (Figure 2D) to exclude the tumor heterogeneity, both TNBC tissues and cell lines dominantly expressed *IL1A*, *IL1B*, *IL8*, and *IL32* (Figure 2D).

### *IL27RA* is predominantly expressed in human BL-BC subtype and TNBC cells

**Figure 3:**
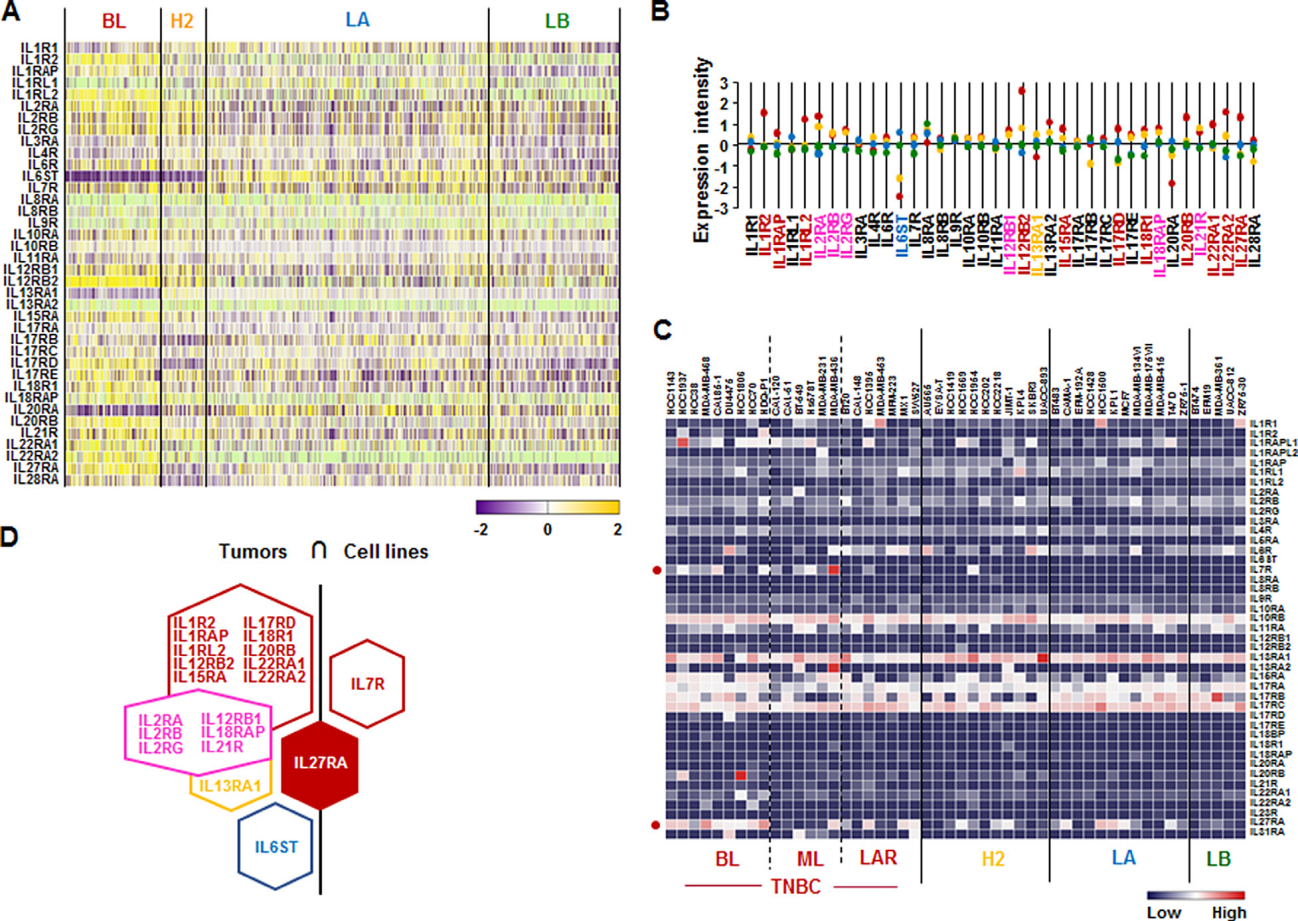
Interleukin receptor superfamily signatures in BC tissues and cell lines (**A**) Heatmap for IL receptor superfamily expression profiles in human BC tissues from TCGA-based dataset using Gitools 2.3.1. (**B**) Statistical analysis of IL receptor superfamily expression intensity in human BC tissues. (**C**) Heatmap for RNA expression levels of IL receptor superfamily based on analysis of GEO dataset (Accession: GSE12777) with 51 human BC cell lines using Gitools 2.3.1. (**D**) Intersection of IL receptor superfamily signature between human BC tissues and cell lines. Red, yellow, blue and green dots specify high expression levels in BL-, HER2 (H2)-, LA- and LB-BC subtypes, respectively. Pink letters specify high expression levels in both BL- and HER2-BC subtypes. ML; mesenchymal-like, LAR; luminal androgen receptor and TNBC; triple-negative breast cancer.

We also assessed the IL receptor superfamily signature in human breast tumor tissues and cell lines. Analysis based on TCGA-based dataset for human BC tissues revealed that BL-BC subtype representing TNBC highly expressed the following receptors: *IL1R2*, *IL1RAP*, *IL1RL2*, *IL12RB2*, *IL15RA*, *IL17RD*, *IL18R1*, *IL20RB*, *IL22RA1*, *IL22RA2*, and *IL27RA* (Figure 3A and 3B). HER2-BC subtype highly expressed *IL13RA1* and LA-BC subtype highly expressed *IL6ST* (Figure 3A and 3B). Both BL- and HER2-BC subtypes highly expressed the following receptors: *IL2RA*, *IL2RB*, *IL2R6*, *IL12RB1*, *IL18RAP* and *IL21R* (Figure 3A and 3B). However, only *IL7R* (BL- and ML-TNBC) and *IL27RA* (BL-TNBC) are highly expressed on human cell lines (Figure 3C and Supplementary Figure 2). Based on the intersection of interleukin receptor superfamily signature between human BC tissues and cell lines (Figure 3D) to exclude the tumor heterogeneity, *IL27RA* was predominantly expressed in both TNBC tissues and cell lines (Figure 3D).

### *CD70* and *TNFSF9* are predominantly expressed in human BL-BC subtype and TNBC cells

**Figure 4:**
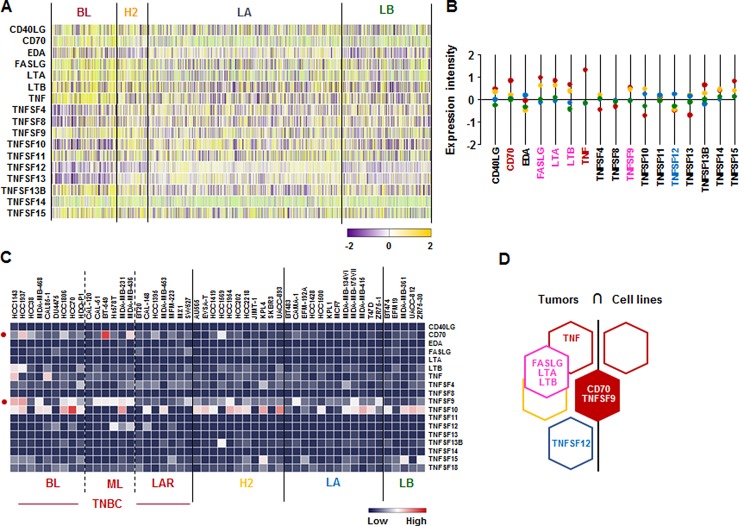
TNF superfamily signatures in BC tissues and cell lines (**A**) Heatmap for TNF superfamily expression profiles in human BC tissues from TCGA-based dataset using Gitools 2.3.1. (**B**) Statistical analysis of TNF superfamily expression intensity in human BC tissues. (**C**) Heatmap for RNA expression levels of TNF superfamily based on analysis of GEO dataset (Accession: GSE12777) with 51 human BC cell lines using Gitools 2.3.1. (**D**) Intersection of TNF superfamily signature between human BC tissues and cell lines. Red, yellow, blue and green dots specify high expression levels in BL-, HER2 (H2)-, LA- and LB-BC subtypes, respectively. Pink letters specify high expression levels in both BL- and HER2-BC subtypes. ML; mesenchymal-like, LAR; luminal androgen receptor and TNBC; triple-negative breast cancer.

We checked the signature of TNF superfamily in human breast tumor tissues and cells lines. According to the analysis on TCGA-based dataset for human BC tissues, *CD70* and *TNF* are highly expressed in BL-BC representing TNBC, while *TNFSF12* is highly expressed in LA-BC subtype (Figure 4A and 4B). Both BL- and HER2-BC subtypes highly expressed *FASLG*, *LTA* and *LTB* (Figure 4A and 4B). Moreover, BL- and ML-TNBC human cell lines highly expressed *CD70* and *TNFSF9* (Figure 4C and Supplementary Figure 3). Based on the intersection of TNF superfamily signature between human BC tissues and cell lines (Figure 4D) to exclude the tumor heterogeneity, both TNBC tissues and cell lines dominantly expressed *CD70* and *TNFSF9* (Figure 4D).

### *TNFRSF21* is mainly expressed in human BL-BC subtype and TNBC cells

**Figure 5:**
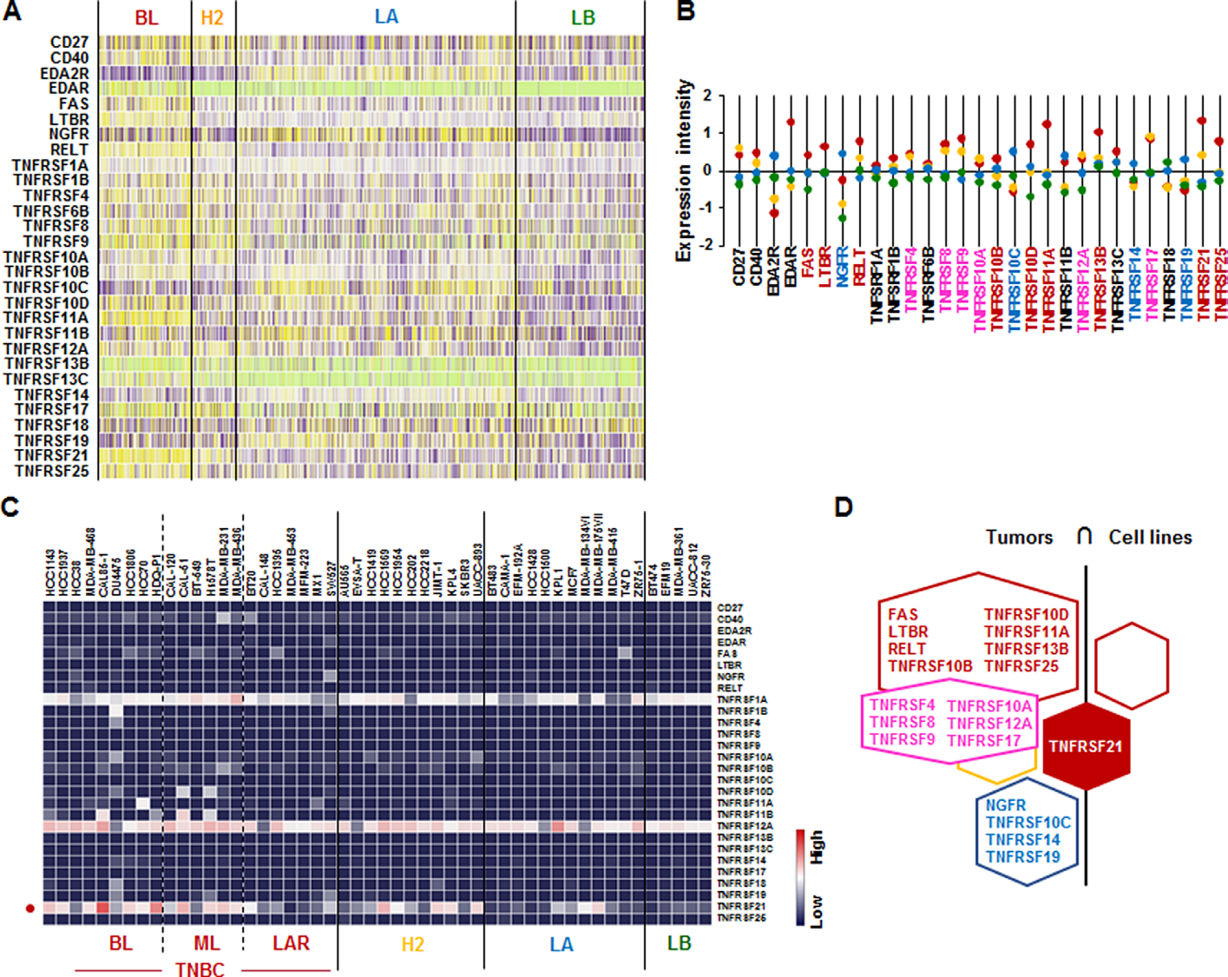
TNF receptor superfamily signatures in BC tissues and cell lines (**A**) Heatmap for TNF receptor superfamily expression profiles in human BC tissues from TCGA-based dataset using Gitools 2.3.1. (**B**) Statistical analysis of TNF receptor superfamily expression intensity in human BC tissues. (**C**) Heatmap for RNA expression levels of TNF receptor superfamily based on analysis of GEO dataset (Accession: GSE12777) with 51 human BC cell lines using Gitools 2.3.1. (**D**) Intersection of TNF receptor superfamily signature between human BC tissues and cell lines. Red, yellow, blue and green dots specify high expression levels in BL-, HER2 (H2)-, LA- and LB-BC subtypes, respectively. Pink letters specify high expression levels in both BL- and HER2-BC subtypes. ML; mesenchymal-like, LAR; luminal androgen receptor and TNBC; triple-negative breast cancer.

We further assessed the TNF receptor superfamily signature in human breast tumor tissues and cells lines. Analysis on TCGA-based dataset for human BC tissues showed that BL-BC subtype representing TNBC highly expressed *FAS*, *LTBR*, *TNFRSF10B*, *TNFRSF10D*, *TNFRSF11A*, *TNFRSF13B*, *TNFRSF21*, and *TNFRSF25* (Figure 5A and 5B). LA-BC subtype highly expressed *NGFR*, *TNFRSF10C*, *TNFRSF14* and *TNFRSF19* (Figure 5A and 5B). Both BL- and HER2-BC subtypes highly expressed *TNFRSF4*, *TNFRSF8*, *TNFRSF9*, *TNFRSF10A*, *TNFRSF12A* and *TNFRSF17* (Figure 5A and 5B). However, our analysis of NCBI GEO dataset on 51 human BC cell lines showed that only *TNFRSF21* is highly expressed in BL- and ML-TNBC cells (Figure 5C and Supplementary Figure 3). Based on the intersection of TNF receptor superfamily signature between human BC tissues and cell lines (Figure 5D) to exclude the tumor heterogeneity, both TNBC tissues and cell lines dominantly expressed *TNFRSF21* (Figure 5D).

### *TLR1* and *TLR2* are predominantly expressed in human BL-BC subtype and TNBC cells

**Figure 6:**
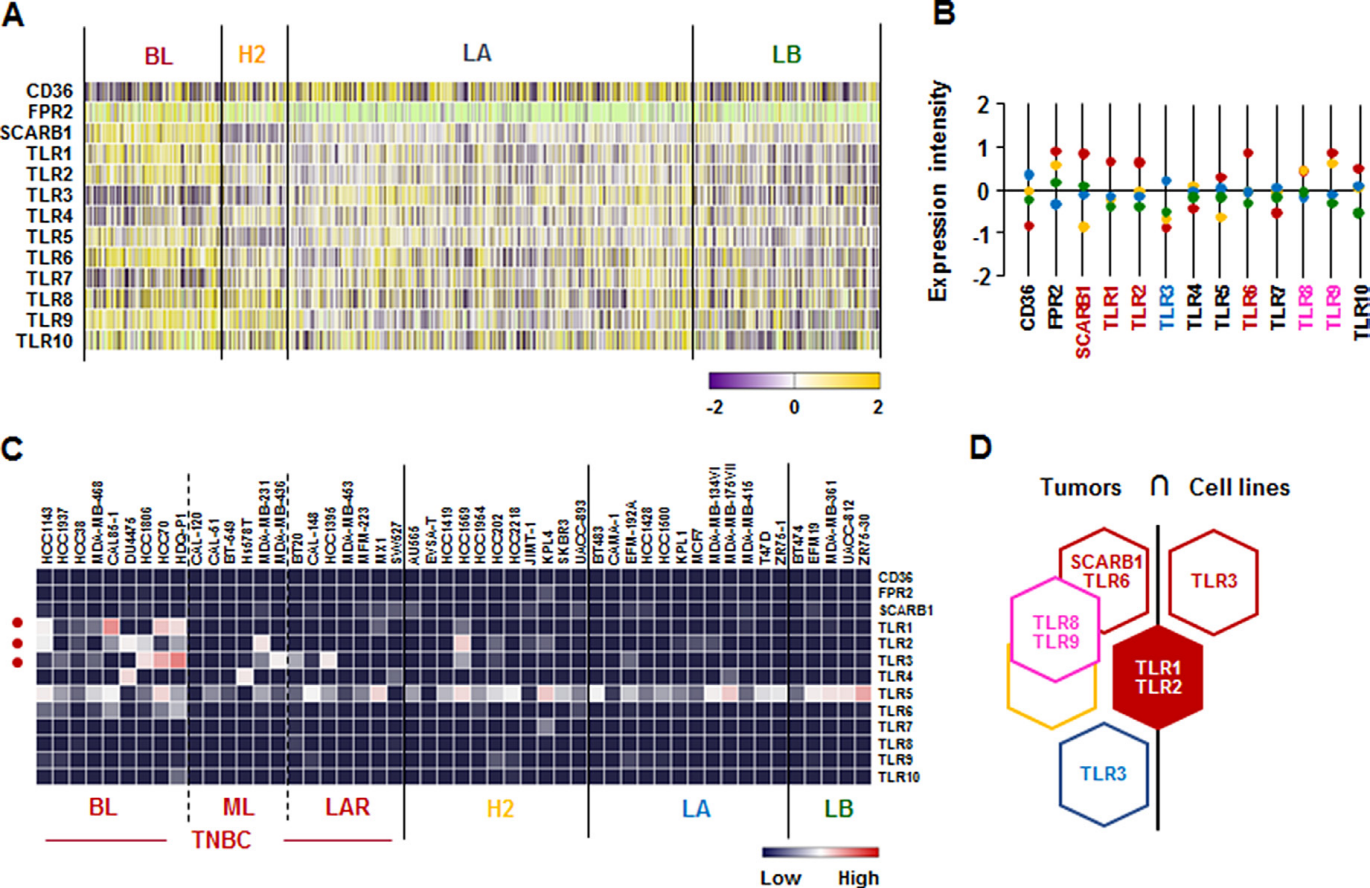
SAA receptor and TLR family signatures in BC tissues and cell lines (**A**) Heatmap for SAA receptor and TLR family expression profiles in human BC tissues from TCGA-based dataset using Gitools 2.3.1. (**B**) Statistical analysis of SAA receptor and TLR family expression intensity in human BC tissues. (**C**) Heatmap for RNA expression levels of SAA receptor and TLR family based on analysis of GEO dataset (Accession: GSE12777) with 51 human BC cell lines using Gitools 2.3.1. (**D**) Intersection of SAA receptor and TLR family signature between human BC tissues and cell lines. Red, yellow, blue and green dots specify high expression levels in BL-, HER2 (H2)-, LA- and LB-BC subtypes, respectively. Pink letters specify high expression levels in both BL- and HER2-BC subtypes. ML; mesenchymal-like, LAR; luminal androgen receptor and TNBC; triple-negative breast cancer.

We investigated the SAA receptor and TLR family signature in human breast tumor tissues and cell lines. SAA has multiple receptors, including the FPR2, the TLRs TLR2 and TLR4, the scavenger receptor SR-BI, and the ATP receptor P2×7 [47]. Analysis on TCGA-based dataset for human BC tissues revealed that the BL-BC subtype representing TNBC highly expressed *SCARB1*, *TLR1*, *TLR2*, and *TLR6*, while LA-BC subtype dominantly expressed *TLR3* (Figure 6A and 6B). Both BL and HER2-subtypes highly expressed *TLR8* and *TLR9* (Figure 6A and 6B). In addition, the analysis of NCBI GEO dataset on human BC cell lines revealed that *TLR1* and *TLR3* were highly expressed in BL-TNBC, while *TLR2* was highly expressed in BL- and ML-TNBC and HER2 subtype (Figure 6C and Supplementary Figure 3). Based on the intersection of SAA receptors and TLR superfamily signature between human BC tissues and cell lines (Figure 6D) to exclude the tumor heterogeneity, *TLR1* and *TLR2* were highly expressed in both TNBC tissues and cell lines (Figure 6D).

### IL-1β augments SAA1 promoter activity via both NF-κB like and consensus sites

**Figure 7:**
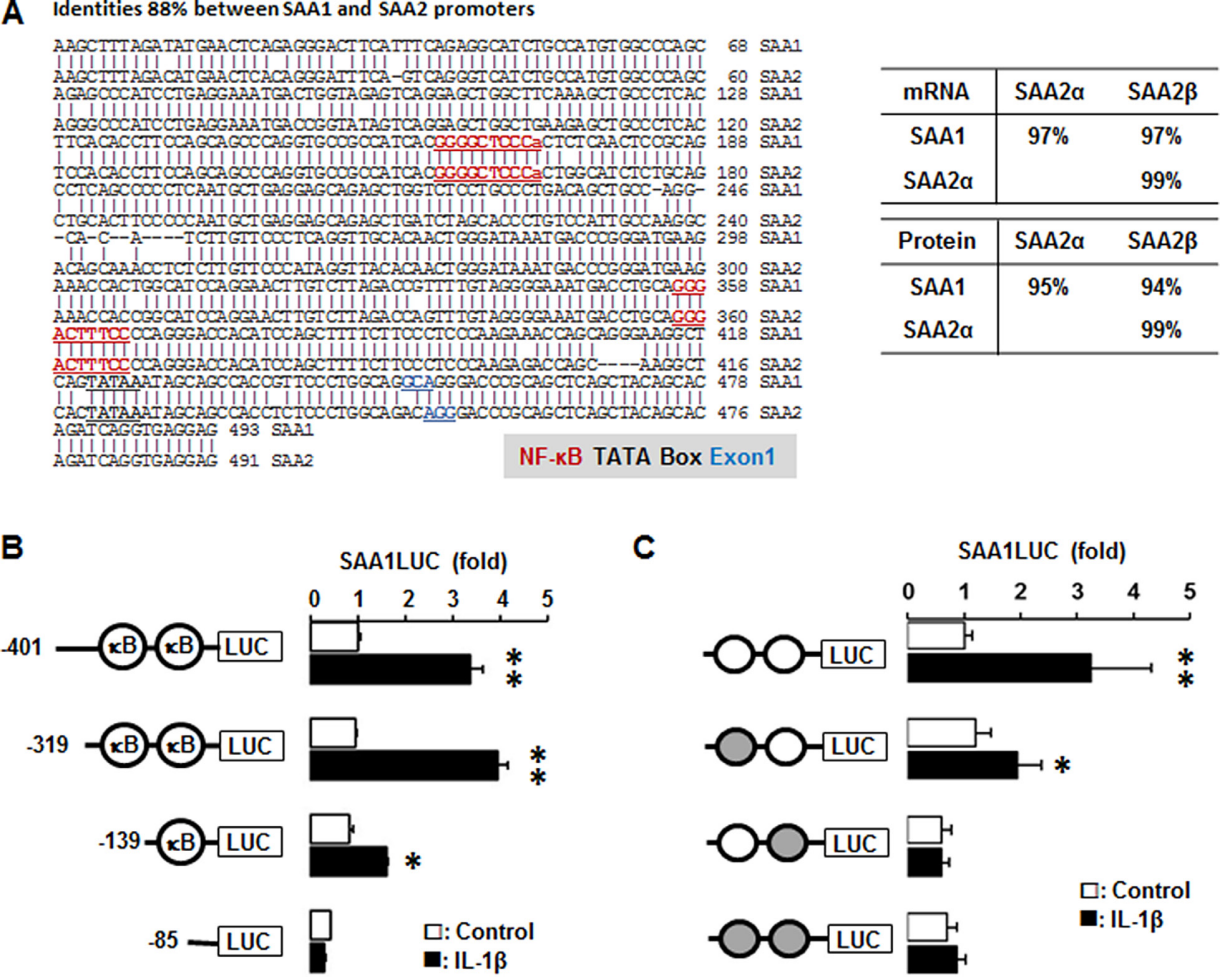
IL-1β increases human SAA1 promoter activity via NF-κB signaling (**A**) DNA sequence and homology of the human SAA1 and SAA2 promoters. (**B**) Effects of IL-1β on luciferase activity in deletion constructs of the SAA1 promoter. After transfection with deletion constructs of SAA1 (SAA1-P401, SAA1-P319, SAA1-P139 and SAA1-P85) luciferase vectors in MDA-MB231 TNBC cells overnight, a luciferase promoter activity assay was performed at post-treatment of IL-1β (10 ng/ml) for 6 h. (**C**) Effects of IL-1β on luciferase activity in NF-κB mutated constructs of the SAA1 promoter. Site-directed mutants were generated from the SAA1-P319LUC using primers with mutant κB-like sites (-287/-278) and κB-consensus site (-95/-86). After transfection with SAA1-P319LUC and its mutant κB-site luciferase vectors in MDA-MB231 TNBC cells overnight, a luciferase promoter activity assay was performed at post-treatment of IL-1β (10 ng/ml) for 6 h. Results were normalized to the protein level and expressed as a fold increase compared to non-treated control. Gray circles indicate κB site mutants. ^*^, ^**^ indicate significant (*p* < 0.05) increase compared to each control, when a Student's-*t* test was analyzed. Also, significant (*p* < 0.05) change exists between ^*^ and ^**^ groups. Representative results are shown from triplicated experiments.

We utilized the Align Sequences Nucleotide and Protein BLAST (https://blast.ncbi.nlm.nih.gov) to check the identities of promoters, mRNAs and proteins of SAA1 and SAA2. The identity of SAA1 and SAA2 is 88% with the same NF-κB like (−287/−278) and consensus (−95/−86) sites (Figure 7A). The identities of mRNAs and proteins for SAA1 and SAA2α are 97% and 95%, SAA1 and SAA2β are 97% and 95%, while the identities of SAA2α and SAA2β are 99% and 99%, respectively (Figure 7A). SAA is restrictedly regulated by NF-κB signaling [21–23] and TNBC cells highly express *IL1A* and *IL1B* (Figure 2) which lead to production of IL-1α and IL-1β. We confirmed IL-1β-induced SAA1 promoter activity in TNBC cells to validate a high level of SAA1/2 in TNBC cells. SAA1-P401, SAA1-P319 and SAA1-P139 deletions were induced by IL-1β, whereas SAA1-P85 without κB sites was not induced (Figure 7B). The SAA1-P401 and SAA1-P319 containing κB-like and consensus sites have a similar induction level, while SAA1-P139 containing only the κB-consensus site has a lower induction level (Figure 7B). This result suggests that both κB-like and consensus sites is critical in regulating IL-1β-induced SAA1 promoter activity in TNBC cells. Based on SAA1-P319LUC (−319/+43) promoter generated previously [23], we further mutated each κB-site in the promoter to investigate which NF-κB sites could be critical in regulating IL-1β-induced SAA1 promoter activity. IL-1β fully induced SAA1-P319 promoter activity, while all of mutants abrogated IL-1β-induced effects (Figure 7C). The mutation of κB-like site at −279/−288 region abrogated IL-1β-induced SAA1 promoter activity without decreasing the basal activity, while the mutation of κB-consensus site at −97/−87 region abrogated both basal and IL-1β-induced activity (Figure 7C). The κB-consensus site at the proximal region −97/−87 appeared to be more critical site in regulating SAA1 even by eliminating IL-1β-induced SAA1 promoter activity, compared to the distal κB-like site (Figure 7C).

### TLR1/2-mediated signaling is involved in regulating IL-1β-induced SAA and SAA1-induced CXCL8 promoter activity in TNBC cells

**Figure 8:**
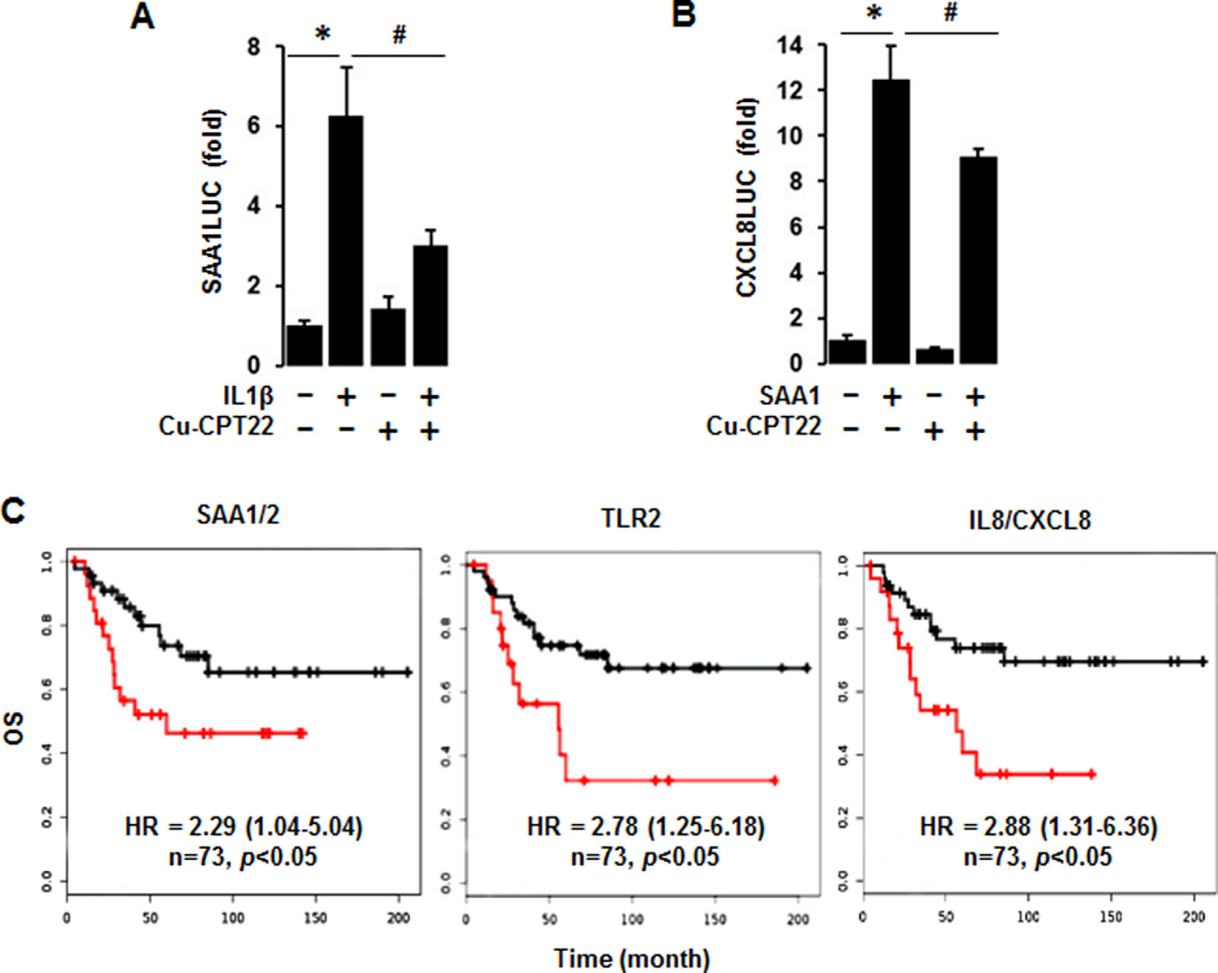
Abrogated effects of Cu-CPT22, a TLR1/2 inhibitor, on IL-1β-induced SAA1 and SAA1-induced CXCL8 promoter activities and overall survival (OS) of SAA1/2, TLR2 and CXCL8 expression levels (**A**) Effect of Cu-CPT22, a TLR1/2 inhibitor, on IL-1β-induced SAA1 promoter activity. After transfection with SAA1-P319 luciferase vectors in MDA-MB231 TNBC cells overnight, a luciferase promoter activity assay was performed at post-treatment of IL-1β (10 ng/ml) for 6 h with a pre-treatment of Cu-CPT22 (1 μM) for 0.5 h. (**B**) Effect of Cu-CPT22, a TLR1/2 inhibitor, on SAA1-induced CXCL8/IL8 promoter activity. After transfection with human CXCL8 promoter (-322/+10) luciferase vectors in MDA-MB231 TNBC cells overnight, a luciferase promoter activity assay was performed at post-treatment of recombinant human SAA1 (500 ng/mL) for 6 h with a pre-treatment of Cu-CPT22 (1 μM) for 0.5 h. Results were normalized to the protein level and expressed as a fold increase compared to non-treated control. *, # indicate significant (*p* ≤ 0.05) increase and decrease, respectively, when ANOVA test was analyzed. Representative results are shown from triplicated experiments. (**C**) Kaplan-Meier OS for SAA1/2, TLR2 and CXCL8 in ML-TNBC patients (*n* = 73). The black and red lines indicate low and high expression levels, respectively.

Because both *TLR1* and *TLR2* among the SAA receptors are highly expressed in TNBC cells (Figure 6), we utilized Cu-CPT22, antagonist of both TLR1 and TLR2, to confirm the involvement of TLR1/2 in IL-1β-induced SAA1 promoter activity. Cu-CPT22 significantly reduced IL-1β-induced SAA1 promoter activity in MDA-MB231 cells (Figure 8A). IL-8/CXCL8, a well-known proinflammatory chemokine [48], were highly expressed in human BL-BC tissues and TNBC cells (Figure 2). Using the previously generated CXCL8 promoter [48], we investigated if SAA1 could induce CXCL8 promoter activity. SAA1 fully induced the CXCL8 promoter activity in MDA-MB231 cells (Figure 8B). Furthermore, Cu-CPT22 partially reduced SAA1-induced CXCL8 promoter activity (Figure 8B). In addition, we analyzed coefficient of determination (R^2^) between *SAA1/2* and TNBC-dominant IL and TNF subfamilies in 51 human BC cell lines as follows: R^2^ = 0.86 with *IL1A*, R^2^ = 0.88 with *IL1B*, R^2^ = 0.59 with *CXCL8*, R^2^ = 0.62 with *IL32*, R^2^ = 0.08 with *IL27RA*, R^2^ = 0.017 with *CD70*, R^2^ = 0.11 with *TNFSF9*, R^2^ = 0.027 with *TNFRSF21*, R^2^ = 0.002 with *TLR1*, and R^2^ = 0.002 with *TLR2* (Supplementary Figure 4). Furthermore, we evaluated Kaplan-Meier overall survival (OS) for *SAA1/2*, *TLR2* and *IL8/CXCL8* which were highly expressed in ML-TNBC. The high expression levels of *SAA1/2* (HR: 2.29, 95% CI: 1.04–5.04), *TLR2* (HR: 2.78, 95% CI: 1.25–6.18) and *IL8/CXCL8* (HR: 2.88, 95% CI: 1.31–6.36) were associated with poor OS in ML-TNBC patients (Figure 8C).

## DISCUSSION

A main finding in this study is that interaction of SAA and proinflammatory cytokines is potentiated in TNBC compared to other BC subtypes, probably leading to inflammatory tumor environment followed by cancer progression and a high mortality. Among expression profiles of APPs, SAA1/2 is emerging as a novel biomarker in TNBC. SAA is an acute-phase protein known to mediate proinflammatory response and generated primarily in the liver. Serum of healthy donors expresses SAA at relatively low levels [49]. SAA protein levels were higher in patients with ER-negative breast tumors compared to those with ER-positive [50], supporting our findings of higher levels of *SAA1/2* in TNBC (Figure 1 and Supplementary Figure 1). High levels of SAA were related to survival time of patients less than one year with breast invasive ductal carcinoma as a useful marker in BC recurrence [51]. Elevated SAA was associated with reduced OS in BC [52]. On the other hand, SAA was linked to poor recurrence-free survival in BC but not OS [53]. Our results indicate that SAA is associated with poor OS in ML-TNBC subtype (Figure 8C) but not all of BC. Interestingly, elevated SAA in tumor-associated macrophage and breast tumor cells was associated with both lymphovascular invasion and lymph node metastasis [53]. In a BC mouse model, ectopic expression of SAA1 or SAA3 in tumor cells potently promoted widespread metastasis [54]. SAA in stages II, III and IV BC patients had a higher value compared to those of the healthy, benign and stage I groups. Also, BC patients with lymph node metastasis or distant metastasis were found to have significantly higher SAA levels [55]. These findings indicate a metastatic effect of SAA in BC, probably contributing to aggressiveness of TNBC which highly expresses SAA (Figure 1 and Supplementary Figure 1). The expression of SAA is restrictedly regulated by proinflammatory cytokines, such as IL-1, IL-6 and TNF [21–23, 56]. Because *IL1A*, *IL1B*, *IL8/CXCL8*, and *IL32* are highly expressed in TNBC (Figure 2 and Supplementary Figure 2), these cytokines can be directly involved in upregulating SAA in TNBC. TNBC tumor irradiation in a mouse model significantly increased the plasma level of IL-1β, which was associated with lung metastases [57]. The basal levels of IL-1β were higher in TNBC cells compared with non-TNBC cells and IL-1β increased invasiveness of TNBC cells [58]. IL-1β also significantly increases IL-8/CXCL8 in TNBC cells [59]. IL-1β was found to induce SAA1 promoter activity in an NF-κB-dependent manner in TNBC cells (Figure 7) and SAA was able to induce IL8/CXCL8 promoter activity (Figure 8B), indicating that interaction of SAA and proinflammatory cytokines could enhance inflammatory burden in TNBC. IL-8/CXCL8 is a blood biomarker for TNBC, significantly increased in TNBC cells compared to non-TNBC, and increase the invasiveness and growth of TNBC cells [60–62]. IL-8/CXCL8 was associated with short disease-free survival and OS in TNBC [60, 63], supporting poor OS to high levels of IL8 in ML-TNBC subtype (Figure 8C). IL-32 increased migration and invasion capacities of TNBC cells [64] and promoted TNBC cell growth [65]. In addition, IL-32 is a potential immunotherapy target antigen in HLA-A2-positive TNBC [66]. Based on these results, a high level of *IL32* in TNBC (Figure 2) may be involved in aggressiveness of TNBC despite unclear direct relationship between SAA and IL32. Although *IL27RA* is found to be predominantly expressed in BL-TNBC (Figure 3 and Supplementary Figure 2), a functional role of IL27RA in BC is poorly understood at this point. CD70 expression levels were significantly higher in BL-BC compared to LA [67], supporting our findings of high levels of *CD70* in TNBC (Figure 4 and Supplementary Figure 3). A functional role of CD70 in BC is poorly understood at this point. TNFSF9 (4-1BB/CD137) antibody favored the propagation of CD8+ tumor-infiltrating lymphocytes (TILs) from TNBC tumors, being capable of cytotoxic functions [68]. This indicates that high levels of TNFSF9 in TNBC (Figure 4 and Supplementary Figure 3) might inhibit CD8+TILs to reduce cytotoxicity, leading to enhanced aggressiveness of TNBC. TNFRSF21 (DR6) level is known to be increased in a grade-dependent manner in BC [69], although the functional role of TNFRSF21 in TNBC is still unclear.

Based on the correlations of APPs with both dominant IL and TNF superfamily in TNBC found by the present studies and other studies, the interrelationship between SAA, IL-1 and IL8/CXCL8 appears to be a main driver in aggressiveness of TNBC. IL-1β-induced SAA1 promoter activity is critically involved in NF-κB-mediated pathway (Figure 7A and 7B) which upregulates IL8/CXCL8 [48]. IL-8/CXCL8 secretion by SAA correlates with NF-κB activation through FPRL1/LXA4R, a G protein-coupled receptor, in neutrophils [70]. SAA induced IL-8 in monocytes and dendritic cells [71] and peripheral blood mononuclear cells (PBMCs) [72]. SAA1 induced IL-8/CXCL8 via TLR4-mediated NF-κB signaling in human bone marrow-derived mesenchymal stem cells (hMSCs) [73]. In addition, SAA released TNF, IL-1β and IL-8 in neutrophils [16]. Although *TLR2* among multiple receptors of SAA cells are highly expressed in TNBC (Figure 6), the interrelation between TLR2 and TNBC is poorly understood. We demonstrate that IL-1β-induced SAA1 and SAA1-induced CXCL8 promoter activities are partially mediated by TLR2 signaling in MDA-MB231 cells (Figure 8A and 8B). Other studies have also shown that SAA could activate NF-κB via TLR2-mediated pathway in HeLa cells and mouse macrophages [72, 73]. TLR2 is expressed in normal mammary epithelia and inhibition of TLR2 reduces growth of human BC cells [74]. Expression of TLR2 was increased in circulating tumor cell-positive patients [75]. TLR2 was highly expressed by two-fold in mammary cancer stem cells and inhibition of TLR2 signaling impaired *in vitro* mammosphere generation in MDA-MB-231 cells [76]. TLR2 mediates invasion by activating NF-κB in MDA-MB-231 cells [77]. Based on these findings, high levels of TLR2 in TNBC (Figure 6) might be closely involved in aggressiveness of TNBC, presenting poor OS to TLR2 in ML-TNBC (Figure 8C). Furthermore, SAA is known to activate the NF-κB signaling pathway in TLR2-dependent manner in other model systems [78–85]. Coefficient of determination indicates that *SAA1/2* are associated with proinflammatory cytokines such as *IL1* and *IL8/CXCL8* in human BC cell lines (Supplementary Figure 4).

In conclusion, IL-1-induced SAA could predispose proinflammatory tumor microenvironment in TNBC, leading to aggressiveness of TNBC followed by a higher mortality.

## MATERIALS AND METHODS

### Reagents

Recombinant human (rh) proteins and inhibitors were purchased as follows: IL-1β from Life Technologies (Carlsbad, CA, USA) and Apo-SAA1 from Peprotech Inc. (Rocky Hill, NJ, USA) and Cu-CPT22 (TLR1 and TLR2 inhibitor) from MilliporeSigma (St. Louis, MO, USA). Antisense and sense oligonucleotides were obtained from Eurofins MWG Operon (Huntsville, AL, USA). Lipofectamine 2000 and all liquid culture media were acquired from Invitrogen (Grand Island, NY, USA). The Luciferase Reporter Assay System was obtained from Promega (Madison, WI, USA).

### Data analysis from GEO and the TCGA datasets

Data analysis was performed using publicly available microarray data sets deposited in NCBI-GEO (http://www.ncbi.nlm.nih.gov/geo/) database under accession number GSE12777. Raw microarray data for APPs, IL family and TNF superfamily were RNA expression levels prepared from 51 human breast cancer cell lines. The basal acute-phase proteins, cytokines and their receptors expression levels were determined by global gene expression profiling of BC cell lines, while molecular subtyping was determined using gene expression and HER2 status by fluorescent *in situ* hybridization. We utilized Gitools 2.3.1 (http://www.gitools.org) based on Oracle Java 7, an open-source tool to perform Genomic Data Analysis and Visualization as interactive heat-maps [86]. Breast invasive carcinoma dataset for TCGA individual projects was used for BC subtypes as follows: *n* = 140 for BL-BC, *n* = 67 for HER2-BC, *n* = 419 for LA-BC and *n* = 192 for LB-BC patients (http://www.gitools.org/datasets/tcga).

### Construction of the SAA1 promoter, its deletion constructs and the SAA1 κB-site mutants

Human SAA1 (−490/+43) promoter was generated as described previously [23]. The following primers were designed: 5′-GGG ATT ATA GGA GTG AGC CAC-3′ for sense and 5′-CTC CTC ACC TGA TCT GTG CTG-3′ for antisense. The PCR was performed for 35 cycles at 94°C for 1 min, 58°C for 1 min and 74°C for 1 min, followed by a final extension at 74°C for 10 min. The amplified SAA1 DNA fragment was then subcloned into pGEM-T easy vector (Promega, Madison, WI). Deletion constructs of pGL4.12 Luciferase Reporter Vector were produced from the SAA1 DNA fragment inserted in pGEM-T Easy Vector under the same PCR conditions using the following primers containing the XhoI and Hind III sites: : 5′-TAA CTC GAG ATC TGC CAT GTG GCC CAG CAG-3′ for SAA1-P401, 5′-TAA CTC GAG ACA CCT TCC AGC AGC CCA GGT-3′ for SAA1-P319, 5′-GCA CTC GAG CCA GGA ACT TGT CTT AGA CCG-3′ for SAA1-P139, and 5′-TAC CTC GAG CCA GGG ACC ACA TCC AGC TTT-3′ for SAA1-P85.

We found two κB sites in SAA1 promoter and generated mutant constructs by mutating each κB site. Primers for mutation of κB-consensus site (lowercase) were designed as follows: 5′-TGA CCT GCA aGG ACT TTC tCC AGG GAC CAC-3′ for −104/−74 and κB-like site; 5′-TGC CGC CAT CAC aGG GCT CCt ACT CTC AAC-3′ for −299/−269. The mutation of κB sites was performed by PCR-based mutagenesis using a site-directed mutagenesis kit according to manufacturer's instructions (Stratagene, La Jolla, CA). The mutant constructs of the SAA1 promoter were confirmed by DNA sequencing analysis. Human CXCL8 promoter (−322/+10) was generated as described previously [48].

### Transient transfection and luciferase assay

TNBC MDA-MB231 cells at approximately 50% confluency in 24-well plates were washed once with fresh media without additives and then transiently transfected with SAA1 constructs and κB-site mutants for 24 h at 37°C using Lipofectamine solution. Transfected cells were treated as outlined in Results and incubated for 6 h. After rinsing cells with cold 1X PBS and adding lysis buffer (Promega, Madison, WI), cell lysates mixed with Bright-Glo^TM^ Assay Reagent (Promega, Madison, WI) were used for determination of luciferase activity using a microplate luminometer. Luciferase activity, expressed as relative light units, was normalized to measured protein levels.

### Overall survival analysis

Kaplan-Meier plotter database was utilized to assess overall survival (OS) using proportional hazards regression to estimate Hazard Ratio (HR) and 95% Confidence Interval (CI) with auto computed cutoff value based on the gene expression levels of *SAA1/2*, *TLR2* and *IL8/CXCL8* from 73 ML-TNBC patients downloaded from GEO (Affymetrix HGU133A and HGU133+2 microarrays) [87].

### Statistics

Data were analyzed by coefficient of determination (R^2^), the paired Student's *t*-test and one-way analysis of variance (ANOVA) as appropriate. If statistical significance (*p* ≤ 0.05) was determined by ANOVA, the data were further analyzed by Tukey's pairwise comparisons to detect specific differences between treatments.

## SUPPLEMENTARY MATERIALS FIGURES



## Abbreviations

ANOVA: one-way analysis of variance
APPs: acute-phase proteins
BC: breast cancer
BL: basal-like
CI: Confidence Interval
EMT: epithelial-to-mesenchymal transition
ER: estrogen receptor
GEO: gene expression omnibus
HER2: epidermal growth factor receptor 2
hMSCs: human bone marrow-derived mesenchymal stem cells
HR: Hazard Ratio
IL: interleukin
LA: luminal A
LAR: luminal androgen
LB: luminal B
ML: mesenchymal-like
NCBI: National Center for Biotechnology Information
NF: nuclear factor
OS: overall survival
PBMCs: peripheral blood mononuclear cells
PR: progesterone receptor
SAA: serum amyloid A
TCGA: The Cancer Genome Atlas
TILs: tumor-infiltrating lymphocytes
TNBC: triple negative breast cancer
TNF: tumor necrosis factor
