# Supplementary material for: Serum amyloid A predisposes inflammatory tumor microenvironment in triple negative breast cancer
Source: Oncotarget. 2019 Jan 11;10(4):511–26. doi: 10.18632/oncotarget.26566 (PMC6355188; doi:10.18632/oncotarget.26566)
Supplement: Supplementary file 1 [file oncotarget-10-511-s001.pdf]

Serum amyloid A predisposes inflammatory tumor microenvironment in triple negative breast cancer

SUPPLEMENTARY MATERIALS

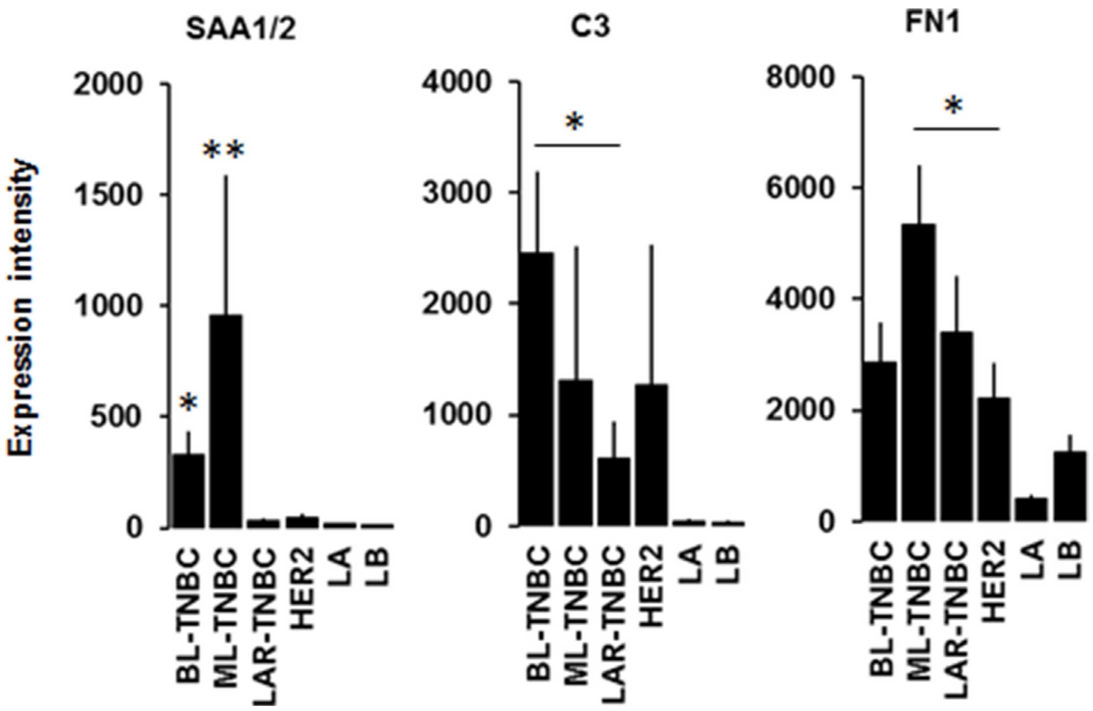

Supplementary Figure 1: Statistical analysis of *SAA1/2*, *C3* and *FN1* expression intensities based on GEO dataset (Accession: GSE12777) with 51 human BC cell lines. BL; basal-like, ML; mesenchymal-like, LAR; luminal androgen receptor and TNBC; triple-negative breast cancer. *, ** indicate significant ($p < 0.05$) increase compared to other subtypes, when a Student's-*t* test was analyzed. Also, significant ($p < 0.05$) change exists between * and ** groups.

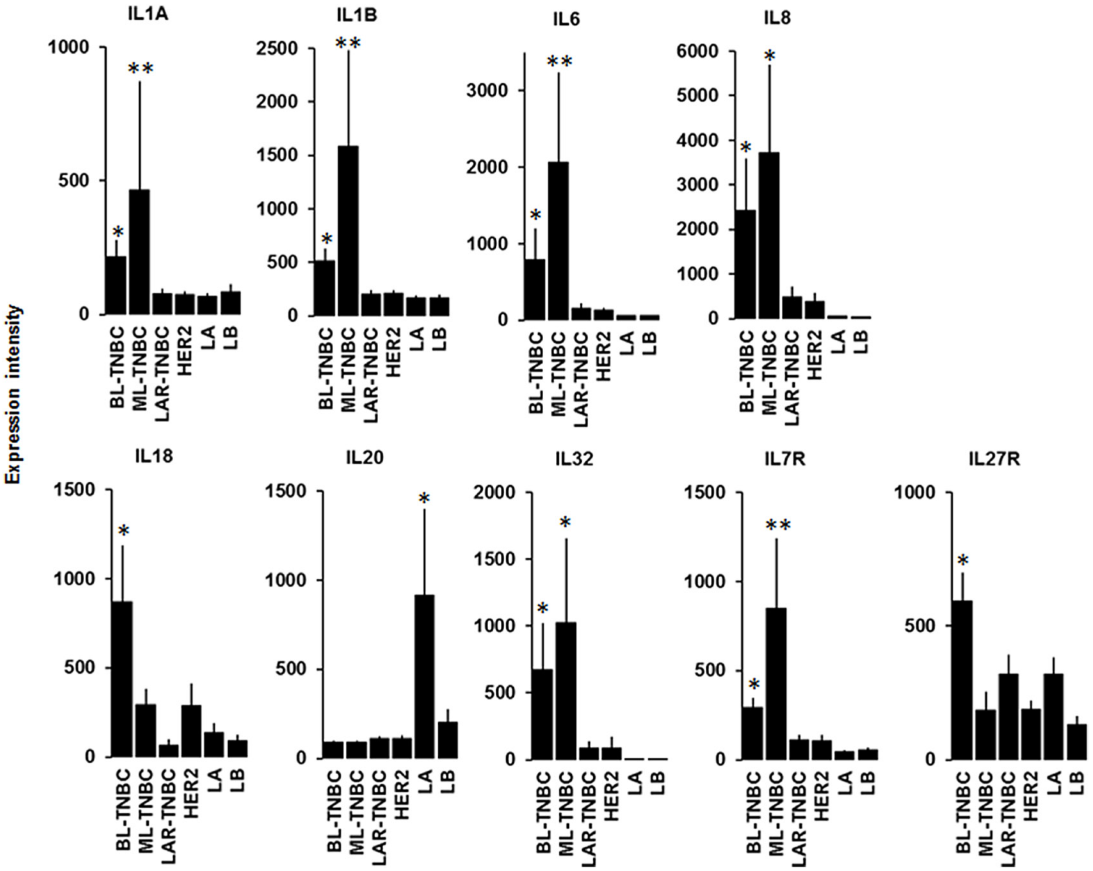

Supplementary Figure 2: Statistical analysis of *IL1A*, *IL1B*, *IL6*, *IL8*, *IL18*, *IL20*, *IL32*, *IL7R* and *IL27R* expression intensities based on GEO dataset (Accession: GSE12777) with 51 human BC cell lines. BL; basal-like, ML; mesenchymal-like, LAR; luminal androgen receptor and TNBC; triple-negative breast cancer. *, ** indicate significant ($p < 0.05$) increase compared to other subtypes, when a Student's-t test was analyzed. Also, significant ($p < 0.05$) change exists between * and ** groups.

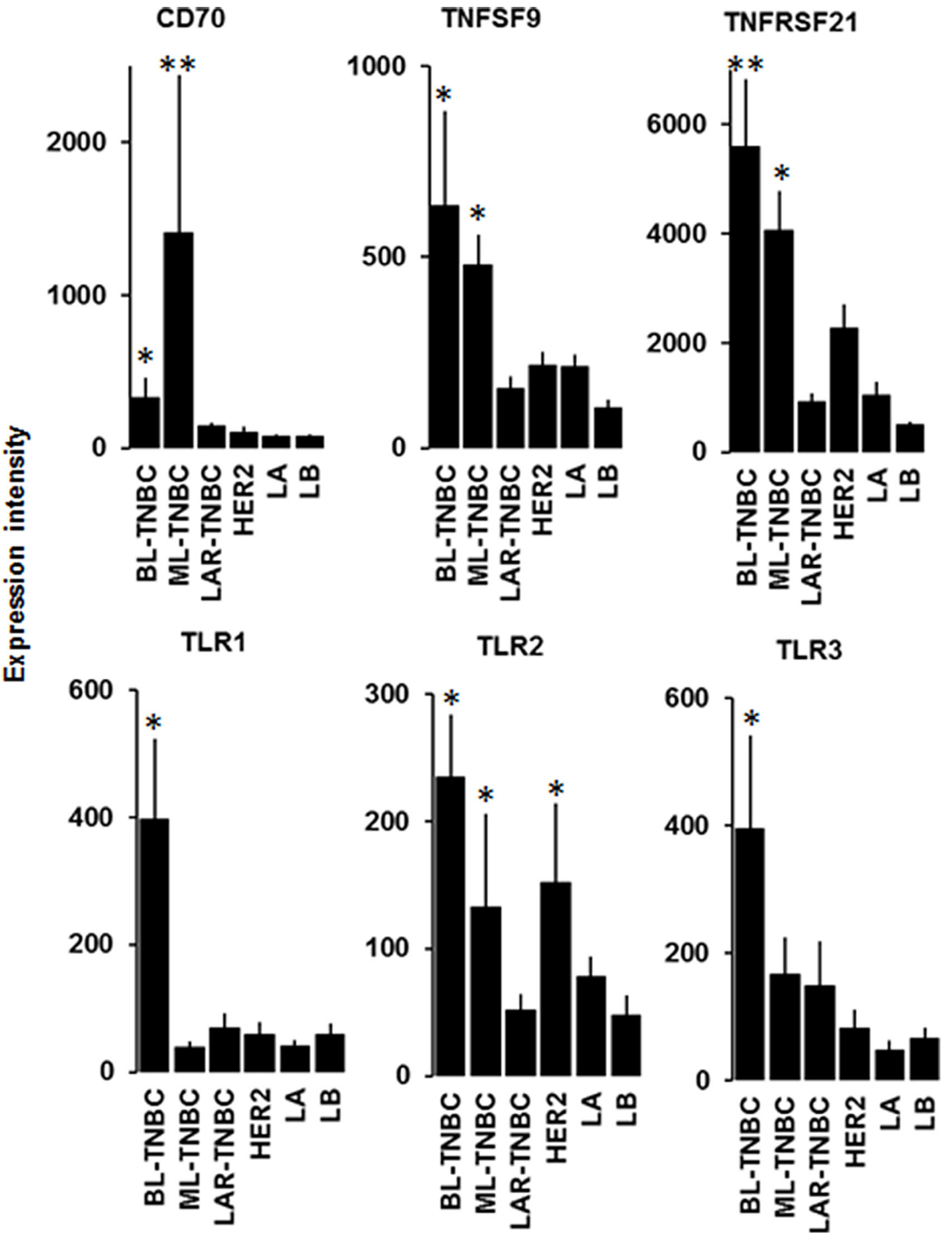

Supplementary Figure 3: Statistical analysis of *CD70*, *TNFSF9*, *TNFRSF21*, *TLR1*, *TLR2* and *TLR3* expression intensities based on GEO dataset (Accession: GSE12777) with 51 human BC cell lines. BL; basal-like, ML; mesenchymal-like, LAR; luminal androgen receptor and TNBC; triple-negative breast cancer. *, ** indicate significant ($p < 0.05$) increase compared to other subtypes, when a Student's-*t* test was analyzed. Also, significant ($p < 0.05$) change exists between * and ** groups.

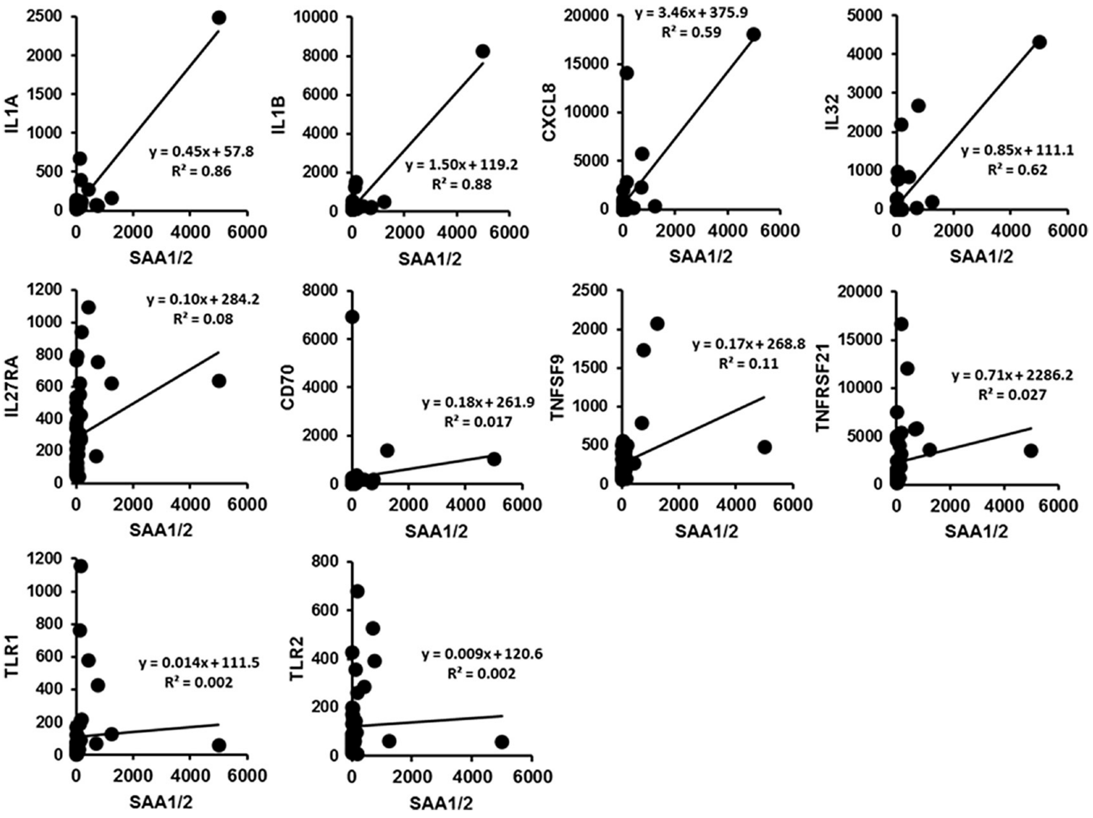

Supplementary Figure 4: Coefficient of determination (R^2) between *SAA1/2* and TNBC-dominant IL and TNF subfamilies such as *IL1A, IL1B, CXCL8, IL32, IL27RA, CD70, TNFSF9, TNFRSF21, TLR1*, and *TLR2* expression intensities based on GEO dataset (Accession: GSE12777) with 51 human BC cell lines.
